# PPIL2 suppression induces cellular senescence and inhibits proliferation in hepatocellular carcinoma *via* c-myc/p21 axis

**DOI:** 10.1016/j.jbc.2026.113109

**Published:** 2026-05-06

**Authors:** Xiaojing Chen, Zhiyao Zhang, Zihan Yan, Xinrui Du, Jihong Wang, Xin Liu, Lanxin Hu, Zhiyan Kuang, Kun He, Jing Zhang, Yunwei Han

**Affiliations:** 1 Department of Oncology, The Affiliated Traditional Chinese Medicine Hospital of Southwest Medical University, Luzhou, China; 2 School of Basic Medical Sciences, State Key Laboratory of Southwestern Chinese Medicine Resources, Chengdu University of Traditional Chinese Medicine, Chengdu, China; 3 Department of Oncology, The Affiliated Hospital of Southwest Medical University, Luzhou, China; 4 Department of Clinical Medicine, Southwest Medical University, Luzhou, China; 5 Clinical Research Institute, The Affiliated Hospital of Southwest Medical University, Luzhou, China; 6 Department of Oncology, The Second Peoples' Hospital of Yibin, Yibin, China

**Keywords:** cell cycle, c-Myc, cellular senescence, hepatocellular carcinoma, PPIL2

## Abstract

Hepatocellular carcinoma (HCC) is the most prevalent primary malignancy of the liver characterized by high mortality rates. While peptidylprolyl isomerase-like two (PPIL2) has been implicated in various malignancies, its functional contribution to HCC progression and the underlying molecular mechanisms remain poorly defined. In this study, we observed significant upregulation of PPIL2 in HCC tissues, which correlated with unfavorable patient prognosis. Functional assays showed that PPIL2 knockdown markedly inhibited HCC cell proliferation by inducing cell cycle arrest and cellular senescence. Mechanistically, PPIL2 depletion led to reduced c-Myc protein levels and a concomitant induction of p21. Critically, the tumor-suppressive effects induced by PPIL2 silencing were partially rescued upon c-Myc overexpression, confirming that PPIL2 exerts its oncogenic function predominantly through a c-Myc-dependent pathway. Furthermore, PPIL2 deficiency effectively hindered tumor growth in a xenograft mouse model. Collectively, these findings identify the PPIL2/c-Myc/p21 axis as a critical regulator of HCC proliferation and senescence, positioning PPIL2 as a promising therapeutic target.

Hepatocellular carcinoma (HCC) accounts for approximately 90% of primary liver cancers and represents the leading cause of cancer-related mortality (1). Despite advancements in multimodal therapies—including surgical resection, molecular-targeted agents, and immunomodulatory approaches—the prognosis for patients with advanced HCC remains dismal due to late-stage diagnosis and the emergence of acquired resistance to first-line tyrosine kinase inhibitors (2, 3). The molecular landscape of HCC is characterized by profound intertumoral heterogeneity and complex oncogenic signaling networks that drive uncontrolled proliferation and metabolic reprogramming (4). Consequently, elucidating the regulatory mechanisms governing HCC progression is imperative for the identification of novel biomarkers and the development of effective therapeutic interventions.

Peptidylprolyl isomerase (cyclophilin)-like 2 (PPIL2), also known as Cyp60 and CYP4, is a U-box-type E3 ubiquitin ligase and a member of the cyclophilin family (5, 6). Beyond its potential role in protein folding, emerging evidence suggests that PPIL2 serves as a pivotal regulator of cancer progression through context-dependent mechanisms. For instance, PPIL2 has been reported to suppress metastasis in breast cancer by modulating cell morphology and the epithelial–mesenchymal transition (EMT) (7). In the context of genomic maintenance, PPIL2 governs DNA resection and homologous recombination by ubiquitinating CtIP (8). Conversely, specific PPIL2 mutations have been linked to a genetic predisposition for lung adenocarcinoma (9). These divergent findings underscore the functional pleiotropy of PPIL2 across different malignancies. However, the clinical significance of PPIL2 and its specific biochemical role in the pathogenesis of HCC remain largely uncharacterized.

In the present study, we demonstrate that PPIL2 is significantly overexpressed in HCC tissues and correlates with poor patient outcomes. Mechanistically, we show that PPIL2 depletion triggers cellular senescence and suppresses HCC cell proliferation by modulating the c-Myc/p21 signaling axis. To our knowledge, this study provides the first evidence identifying PPIL2 as a pro-proliferative driver in HCC, thereby expanding the known functional repertoire of the cyclophilin family. Our findings highlight PPIL2 as a critical regulator of the senescence-proliferation balance and suggest its potential as a promising therapeutic target for HCC.

## Results

### PPIL2 is upregulated in HCC and correlates with poor prognosis

**Figure 1:**
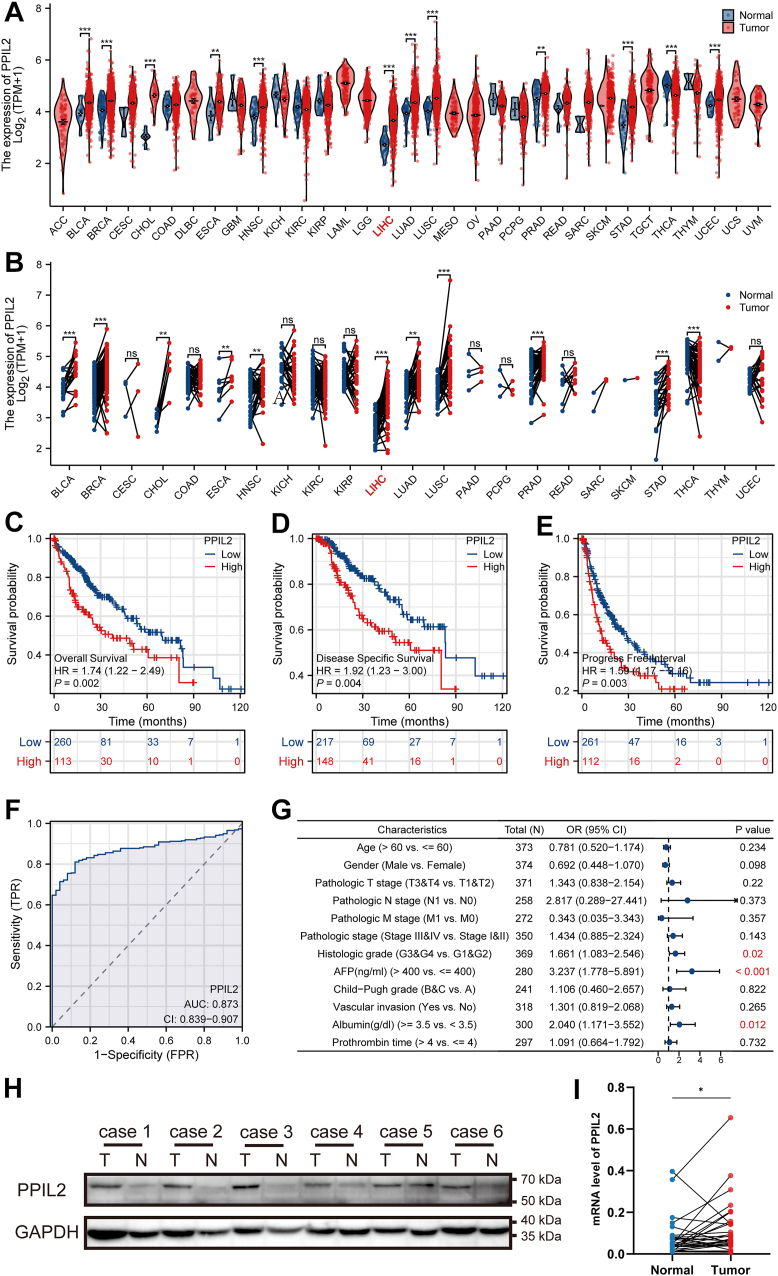
**Upregulation of PPIL2 correlates with malignant phenotypes and poor prognosis in HCC.***A*, PPIL2 expression profiles in 33 human cancers based on TCGA tumor and normal tissue datasets. Wilcoxon rank sum test; ∗∗*p* < 0.01; ∗∗∗*p* < 0.001. *B*, comparative analysis of PPIL2 expression between tumor tissues and paired adjacent non-tumor liver tissues from TCGA cohorts. Wilcoxon signed rank test; ∗∗*p* < 0.01; ∗∗∗*p* < 0.001; ns, not significant. *C–E*, Kaplan–Meier survival curves with risk tables for (*C*) overall survival (OS), (*D*) disease-specific survival (DSS), and (*E*) progression-free interval (PFI) in TCGA-LIHC patients. Patients were stratified into PPIL2 high-expression and low-expression groups using the optimal cut-off value of PPIL2 expression determined by the surv_cutpoint function. Statistical significance was assessed by Cox regression analysis. *F*, receiver–operating characteristic (ROC) curve evaluating the diagnostic efficacy of PPIL2 for distinguishing HCC tumor tissues from normal tissues, with area under the curve (AUC) value indicated (AUC = 0.873). *G*, logistic regression analysis assessing the association between PPIL2 expression and clinicopathological characteristics of HCC, based on 374 HCC cases from the TCGA-LIHC cohort. Odds ratios (OR) and 95% confidence intervals (CI) are indicated. *H*, Western blot analysis of PPIL2 protein expression in human HCC tissues and paired adjacent non-tumor tissues. *I*, quantitative RT-PCR analysis of PPIL2 mRNA expression in 30 pairs of human HCC tissues and matched adjacent normal tissues. Data were normalized to the housekeeping gene GAPDH. Statistical significance was determined by two-tailed paired Student’s *t* test. ∗*p* < 0.05.

To assess the clinical relevance of PPIL2 across human malignancies, we first analyzed transcriptomic data from the TCGA database, encompassing 730 normal and 11,363 tumor samples across 33 cancer types. PPIL2 transcript levels were significantly elevated in 11 malignancies compared with their respective normal tissues, including bladder urothelial carcinoma (BLCA), breast invasive carcinoma (BRCA), cholangiocarcinoma (CHOL), esophageal carcinoma (ESCA), head and neck squamous cell carcinoma (HNSC), liver hepatocellular carcinoma (LIHC), lung adenocarcinoma (LUAD), lung squamous cell carcinoma (LUSC), prostate adenocarcinoma (PRAD), stomach adenocarcinoma (STAD), and uterine corpus endometrial carcinoma (UCEC) (Fig. 1*A*). Conversely, PPIL2 was downregulated in thyroid carcinoma (THCA). This differential expression pattern was further substantiated by paired-tissue analysis, which confirmed PPIL2 upregulation in the 11 aforementioned cancers, suggesting a potential oncogenic role (Fig. 1*B*).

We next focused on the prognostic significance of PPIL2 in HCC. Kaplan–Meier analysis revealed that elevated PPIL2 expression was significantly associated with shortened overall survival (OS), disease-specific survival (DSS), and progression-free interval (PFI) (Fig. 1, *C–E*). The diagnostic efficacy of PPIL2 was validated by ROC analysis, achieving an AUC of 0.873 for tumor-normal discrimination (Fig. 1*F*). Furthermore, logistic regression analysis of 374 HCC cases demonstrated that high PPIL2 expression positively correlated with advanced histologic grades (OR = 1.661, 95% CI 1.083–2.546), elevated AFP levels (OR = 3.237, 95% CI 1.778–5.892), and elevated albumin levels (OR = 2.040, 95% CI 1.171–3.552) (Fig. 1*G*).

To validate these bioinformatic findings, we examined PPIL2 expression in HCC patient samples. Western blot analysis confirmed that PPIL2 protein levels were markedly higher in HCC tissues compared with matched adjacent normal tissues (Fig. 1*H*). Additionally, we evaluated PPIL2 transcription levels, and these analyses again revealed that PPIL2 expression was significantly higher in HCC tissues compared to adjacent normal tissues (n = 30) (Fig. 1*I*). Collectively, these results demonstrate that PPIL2 is frequently overexpressed in HCC and serves as a robust indicator of poor clinical prognosis.

### PPIL2 promotes the HCC cell proliferation

**Figure 2:**
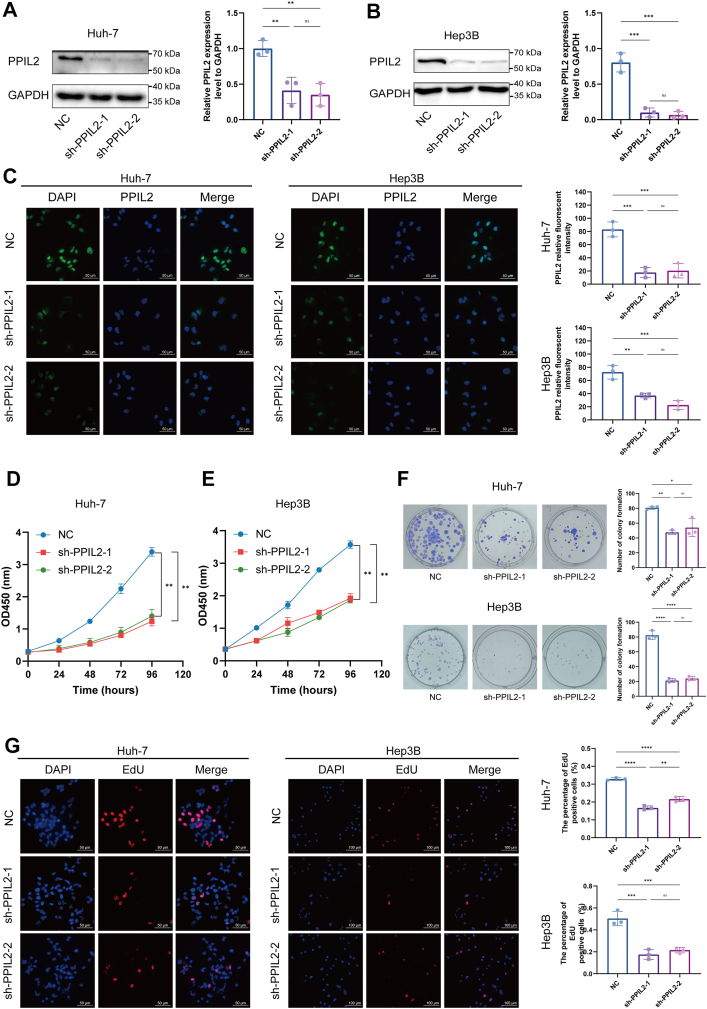
**PPIL2 positively regulates the proliferation of HCC cells *in vitro*.***A* and *B*, Western blot validation of PPIL2 knockdown efficiency in (*A*) Huh-7 and (*B*) Hep3B cells transduced with PPIL2-specific shRNA or non-target control (NC) shRNA. Protein expression was normalized to the housekeeping gene GAPDH. Quantification of blots data represented as mean ± S.D. from three independent biological replicates. Statistical significance was determined by one-way ANOVA with Tukey's multiple comparisons test. ∗*p* < 0.05; ∗∗∗*p* < 0.001; ns, not significant. *C*, immunofluorescence staining of PPIL2 (*green*), and nuclei (DAPI, *blue*) in Huh-7 and Hep3B cells after PPIL2 knockdown, showing PPIL2 subcellular localization and knockdown efficiency. Scale bar: 50 μm. Quantitative analysis of relative PPIL2 fluorescent intensity (normalized to the NC group) is shown as mean ± S.D. from three independent biological replicates. Statistical significance was determined by one-way ANOVA with Tukey's multiple comparisons test. ∗∗*p* < 0.01; ∗∗∗*p* < 0.001; ns, not significant. *D* and *E*, CCK-8 assay evaluating the proliferative capacity of (*D*) Huh-7 and (*E*) Hep3B cells following PPIL2 knockdown. Data are expressed as mean ± S.D. from three independent biological replicates. Statistical significance was determined by two-way ANOVA with Sidak's multiple comparisons test. ∗∗*p* < 0.01. *F*, colony formation assay assessing the long-term clonogenic ability of Huh-7 and Hep3B cells with PPIL2 knockdown. The number of colonies is expressed as the mean ± S.D. from three independent biological replicates. Statistical significance was analyzed by one-way ANOVA with Tukey's multiple comparisons test. ∗*p* < 0.05; ∗∗*p* < 0.01; ∗∗∗∗*p* < 0.0001; ns, not significant. *G*, EdU assay to assess the proliferation capacity of Huh-7 and Hep3B cells after PPIL2 knockdown. Scale bar: 50μm for Huh-7 cells and 100μm for Hep3B cells. The percentage of EdUpositive cells is expressed as the mean ± S.D. from three independent biological replicates. Statistical significance was analyzed by one-way ANOVA with Tukey's multiple comparisons test. ∗∗*p* < 0.01; ∗∗∗*p* < 0.001; ∗∗∗∗*p* < 0.0001; ns, not significant.

The preceding bioinformatic analyses implied PPIL2's potential role in HCC pathogenesis and progression. To verify these observations, we performed loss-of-function experiments in Huh-7 and Hep3B cells using lentiviral-mediated shRNA knockdown, which achieved robust depletion of PPIL2 protein levels (Fig. 2, *A* and *B*). Immunofluorescence analysis confirmed the efficient knockdown and revealed that PPIL2 is predominantly localized within the nucleus (Fig. 2*C*). To evaluate the functional impact of PPIL2, we conducted CCK-8, colony formation, and EdU incorporation assays. CCK-8 analysis demonstrated that PPIL2 knockdown significantly reduced the proliferative capacity of both cell lines (Fig. 2, *D* and *E*). Consistent with these findings, colony formation was markedly impaired (Fig. 2*F*), and EdU assays showed a significant decrease in the proportion of S-phase cells following PPIL2 silencing (Fig. 2*G*). These data indicate that PPIL2 is essential for maintaining HCC cell proliferation.

### PPIL2 is involved in the HCC cell cycle acceleration and proliferative signaling

**Figure 3:**
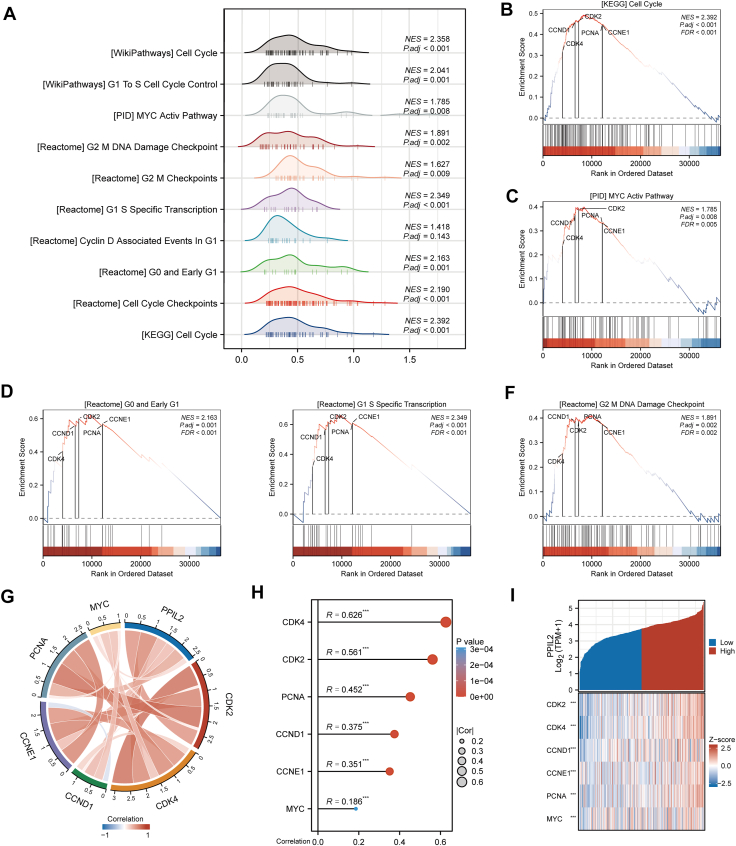
**PPIL2 expression is associated with cell cycle and proliferative signaling activation in HCC *via* transcriptomic analysis.***A*, Joy plot of Gene Set Enrichment Analysis (GSEA) showing significantly enriched cell cycle- and proliferation-related pathways in the PPIL2 high-expression group (vs. low-expression group, stratified by median expression) from the TCGA-LIHC cohort. Bandwidth reflects gene set density, and peak height indicates pathway activation intensity. *B–F*, GSEA enrichment plots for core pathways: (*B*) cell cycle, (*C*) MYC active pathway, (*D*) G0 and early G1 phases, (*E*) G1/S-specific transcription, and (*F*) G2/M DNA damage checkpoint. *G*, Chord diagram illustrating gene correlations at the class level, based on Spearman’s rank correlation between PPIL2 and cell cycle-related genes in HCC. *H*, lollipop plot quantifying Pearson’s correlation coefficients between PPIL2 expression and transcript levels of core cell cycle genes (CDK2, CDK4, CCND1, CCNE1, PCNA, MYC) in HCC. *I*, gene co-expression heatmap showing the correlation between PPIL2 and cell cycle regulatory genes in the TCGA-LIHC cohort.

To elucidate the underlying mechanisms of PPIL2 in promoting HCC cell proliferation, we integrated transcriptomic data from the TCGA-LIHC cohort. Gene Set Enrichment Analysis (GSEA) revealed that high PPIL2 expression is significantly associated with the enrichment of cell cycle-related pathways and Myc signaling (Fig. 3*A*). Notably, key cell cycle regulators, including CDK2, CDK4, CCND1 (Cyclin D1), CCNE1 (Cyclin E1), and PCNA, were identified as core components of these enriched gene sets (Fig. 3, *B–F*). Correlation analysis further demonstrated a strong positive association between PPIL2 and these regulators at the transcript level (Fig. 3, *G–I*), suggesting that PPIL2 may orchestrate a regulatory network that accelerates the HCC cell cycle.

### Knockdown of PPIL2 induces cell cycle arrest in HCC cells

**Figure 4:**
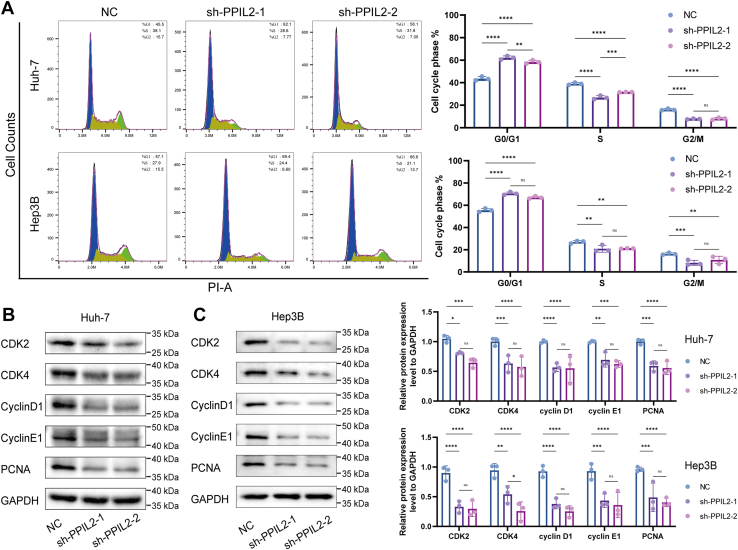
**PPIL2 knockdown induces cell cycle arrest and downregulates key cell cycle regulatory proteins in HCC cells.***A*, flow cytometry analysis of cell cycle distribution in Huh-7 and Hep3B cells with PPIL2 knockdown, showing the proportion of cells in G0/G1, S, and G2/M phases. Data are mean ± S.D. from three independent biological replicates. Two-way ANOVA with Sidak's multiple comparisons test; ∗∗*p* < 0.01; ∗∗∗*p* < 0.001; ∗∗∗∗*p* < 0.0001; ns, not significant. *B* and *C*, Western blot analysis of protein expression of key cell cycle regulators (CDK2, CDK4, Cyclin D1, Cyclin E1, PCNA) in (*B*) Huh-7 and (*C*) Hep3B cells following PPIL2 knockdown. Protein expression was normalized to the housekeeping gene GAPDH. Data are mean ± SD from three independent biological replicates. Statistical significance was determined by two-way ANOVA with Sidak's multiple comparisons test. ∗∗∗*p* < 0.001; ∗∗∗∗*p* < 0.0001; ns, not significant.

To further elucidate the role of PPIL2 in cell cycle regulation, flow cytometry-based cell cycle analysis was performed. PPIL2 knockdown resulted in a marked accumulation of Huh-7 and Hep3B cells in G0/G1 phase with corresponding reductions in S and G2/M phase populations (Fig. 4*A*). Consistent with the cell cycle analysis findings, Western blot analysis revealed significant PPIL2-mediated regulation of key cell cycle proteins. Knockdown of PPIL2 in Huh-7 and Hep3B cells substantially downregulated protein levels of CDK2, CDK4, Cyclin D1, Cyclin E1, and PCNA (Fig. 4, *B* and *C*). These results confirm that PPIL2 is required for efficient cell cycle progression in HCC cells.

### Knockdown of PPIL2 induces cellular senescence in HCC cells

**Figure 5:**
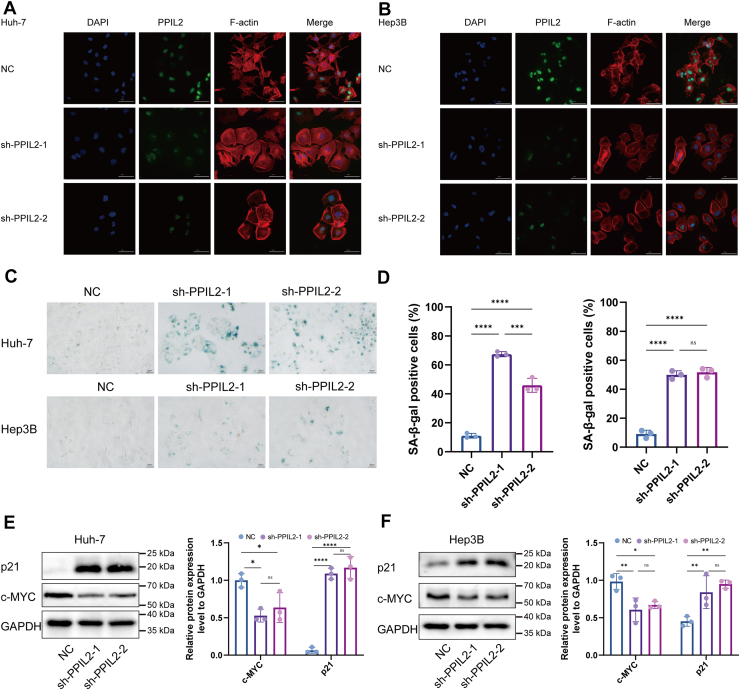
**PPIL2 knockdown induces cellular senescence and modulates the c-Myc/p21 axis in HCC cells.***A* and *B*, immunofluorescence staining of F-actin (*red*), PPIL2 (*green*), and nuclei (DAPI, *blue*) in (*A*) Huh-7 and (*B*) Hep3B cells with PPIL2 knockdown, showing morphological changes of senescent cells (enlarged and flattened cell shape). Scale bar: 50μm. *C*, representative images of SA-β-gal staining in Huh-7 and Hep3B cells after PPIL2 knockdown. Scale bar: 50μm. *D*, quantitative analysis of the proportion of SA-β-gal-positive cells in *panel C*. Data are mean ± SD from three independent biological replicates. Statistical significance was determined by two-way ANOVA with Sidak's multiple comparisons test. ∗∗∗*p* < 0.001; ∗∗∗∗*p* < 0.0001; ns, not significant. *E* and *F*, Western blot analysis of c-Myc and p21 protein expression in (*E*) Huh-7 and (*F*) Hep3B cells following PPIL2 knockdown. Protein expression was normalized to the housekeeping gene GAPDH. Data are mean ± SD from three independent biological replicates. Statistical significance was determined by two-way ANOVA with Sidak's multiple comparisons test. ∗*p* < 0.05; ∗∗*p* < 0.01; ∗∗∗*p* < 0.001; ns, not significant.

Cellular senescence is a state in which cells undergo permanent cell cycle arrest, exhibit increased SA-β-gal activity, and exhibit dysregulated metabolism. Senescent cells also become flattened and enlarged, which are key morphological features of senescence (10). The above results indicate that knockdown of PPIL2 leads to significant cell cycle arrest. Furthermore, we observed similar morphological changes in both Huh-7 and Hep3B cells following PPIL2 knockdown, specifically a noticeable increase in cell size (Fig. 5, *A* and *B*), suggesting the possible induction of cellular senescence. To further validate this phenotype, we conducted SA-β-gal staining assays. The results showed a marked increase in senescent cells (Fig. 5, *C* and *D*), which further supports the conclusion that PPIL2 knockdown can induce cellular senescence.

c-Myc is a key negative regulator of cellular senescence. Its mechanism primarily involves the suppression of p21 transcription to sustain cell proliferation; when c-Myc expression is inhibited, the repression of p21 is lifted, leading to cell cycle arrest and a senescent phenotype (11, 12, 13). Based on GSEA analysis indicating a transcriptional correlation between PPIL2 and the MYC pathway, we further examined their regulatory relationship at the protein level. Experimental results demonstrated that knockdown of PPIL2 significantly reduced c-Myc protein expression, while p21 protein levels were markedly increased. These findings suggest that PPIL2 maintains proliferation by sustaining c-Myc levels and suppressing p21-mediated senescence.

### PPIL2 knockdown triggers HCC senescence *via* inhibition of the c-myc/p21 axis

**Figure 6:**
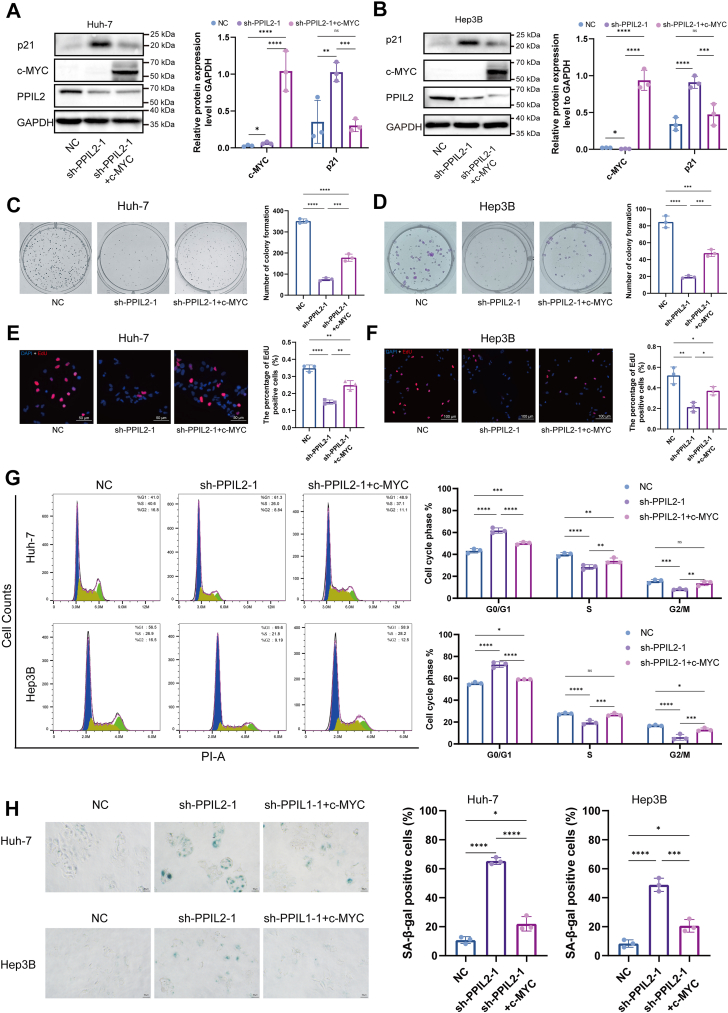
**c-Myc overexpression reverses PPIL2 knockdown-induced proliferation inhibition, cell cycle arrest and cellular senescence in HCC cells.***A* and *B*, Western blot analysis of c-Myc and p21 protein expression in (*A*) Huh-7 and (*B*) Hep3B cells with PPIL2 knockdown alone, or PPIL2 knockdown combined with c-Myc overexpression. Protein expression was normalized to the housekeeping gene GAPDH. Data are mean ± SD from three independent biological replicates. Statistical significance was determined by two-way ANOVA with Sidak's multiple comparisons test. ∗*p* < 0.05; ∗∗*p* < 0.01; ∗∗∗*p* < 0.001; ∗∗∗∗*p* < 0.0001; ns, not significant. *C* and *D*, colony formation assay evaluating the clonogenic ability of (*C*) Huh-7 and (*D*) Hep3B cells under the indicated treatment conditions. The number of colonies is expressed as the mean ± S.D. from three independent biological replicates. Statistical significance was analyzed by one-way ANOVA with Tukey's multiple comparisons test. ∗∗∗*p* < 0.001; ∗∗∗∗*p* < 0.0001. *E* and *F*, EdU assay assessing the proliferative capacity of (*E*) Huh-7 and (*F*) Hep3B cells under the indicated treatment conditions. Scale bar: 50μm for Huh-7 cells and 100 μm for Hep3B cells. The percentage of EdU-positive cells is expressed as the mean ± S.D. from three independent biological replicates. Statistical significance was analyzed by one-way ANOVA with Tukey's multiple comparisons test. ∗*p* < 0.05; ∗∗*p* < 0.01; ∗∗∗*p* < 0.001. *G*, flow cytometry analysis of cell cycle distribution in Huh-7 and Hep3B cells with PPIL2 knockdown alone, or PPIL2 knockdown combined with c-Myc overexpression. Data are mean ± S.D. from three independent biological replicates. Two-way ANOVA with Sidak's multiple comparisons test; ∗*p* < 0.05; ∗∗*p* < 0.01; ∗∗∗*p* < 0.001; ∗∗∗∗*p* < 0.0001; ns, not significant. *H*, representative images of SA-β-gal staining in Huh-7 and Hep3B cells under the indicated treatment conditions, with quantitative analysis of the proportion of SA-β-gal-positive cells. Scale bar: 50 μm. Data are mean ± S.D. from three independent biological replicates. Statistical significance was determined by two-way ANOVA with Sidak's multiple comparisons test. ∗*p* < 0.05; ∗∗∗*p* < 0.001; ∗∗∗∗*p* < 0.0001.

To determine if the effects of PPIL2 are mediated *via* c-Myc, we performed rescue experiments by overexpressing c-Myc in PPIL2-knockdown cells. The induction of p21 by PPIL2 silencing was partially attenuated upon c-Myc overexpression (Fig. 6, *A* and *B*). Moreover, colony-formation and EdU assays indicated that the proliferative capacity was significantly restored upon c-Myc overexpression in PPIL2-deficient HCC cells (Fig. 6, *C–F*). Furthermore, c-Myc overexpression partially reversed the cell cycle arrest induced by PPIL2 knockdown (Fig. 6*G*). As expected, SA-β-gal staining assays showed that the proportion of senescent cells was reduced when c-Myc was overexpressed in PPIL2-knockdown HCC cells (Fig. 6*H*). Collectively, c-Myc overexpression partially re-establishes the oncogenic functions mediated by PPIL2, counteracting the abrogation of cellular senescence and tumor progression resulting from its knockdown in HCC cells.

To exclude potential off-target effects of the shRNA, we performed additional rescue experiments using a Flag-tagged PPIL2 construct in Huh-7 cells (Fig. S1*A*). Functional assays revealed that re-expression of PPIL2 completely reversed the proliferative defects, cell cycle arrest, and increased cellular senescence triggered by PPIL2 knockdown (Fig. S1, *B–E*). At the molecular level, the dysregulated expression of PPIL2 downstream effectors c-Myc and p21 induced by gene silencing was fully normalized to control levels upon PPIL2 rescue (Fig. S1*F*). These data definitively establish that the observed phenotypes are specifically driven by the loss of PPIL2.

### Silencing PPIL2 suppressed tumor growth *in vivo*

**Figure 7:**
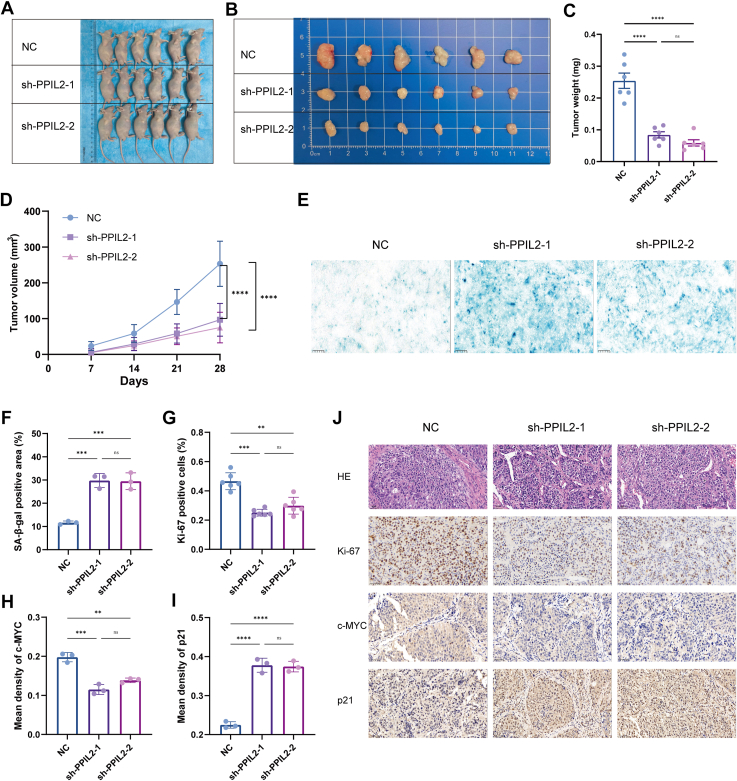
**PPIL2 knockdown inhibits HCC tumor growth and induces cellular senescence *in vivo.****A*, representative images of nude mice bearing subcutaneous HCC xenografts from the NC group and PPIL2 shRNA groups at the experimental endpoint. *B*, representative images of excised subcutaneous tumor specimens collected at the experimental endpoint. *C*, quantitative analysis of final tumor weights from the NC group and PPIL2 shRNA groups. Data are mean ± SD (n = 6 independent biological replicates). Statistical significance was analyzed by one-way ANOVA with Tukey's multiple comparisons test. ∗∗∗∗*p* < 0.0001; ns, not significant. *D*, tumor growth curves recorded every week after injection of tumor cells for the NC group and PPIL2 shRNA groups (n = 6 independent biological replicates). Statistical significance was determined by two-way ANOVA with Sidak's multiple comparisons test. ∗∗∗∗*p* < 0.0001. *E*, representative images of SA-β-gal staining performed on frozen sections of subcutaneous tumor tissues from each group. Scale bar: 50μm. *F*, quantitative analysis of the percentage of SA-β-gal-positive areas in *panelE*. Data are mean ± S.D. from three independent biological replicates. Statistical significance was analyzed by one-way ANOVA with Tukey's multiple comparisons test. ∗∗∗*p* < 0.001; ns, not significant. *G–J*, Histopathological and IHC analysis of tumor tissues: (*G*) H&E staining for tumor cell morphology, and IHC staining for (*H*) Ki67, (*I*) c-Myc, and (*J*) p21 in tumor tissues from the NC and PPIL2 shRNA groups. Scale bar: 50μm. Data are expressed as mean ± SD from three independent biological replicates. Statistical significance was analyzed by one-way ANOVA with Tukey's multiple comparisons test. ∗∗*p* < 0.01; ∗∗∗*p* < 0.001, ∗∗∗∗*p* < 0.0001; ns, not significant.

To further validate our *in vitro* findings, we conducted *in vivo* experiments. A xenograft tumor model was established in nude mice by implanting Huh-7 cells transduced with either non-target control (NC) or PPIL2 shRNA. Representative images of tumor-bearing nude mice and excised subcutaneous tumor tissues are shown in Figure 7*A* and *B*. PPIL2-silenced tumors exhibited significantly reduced weight and volume compared to the NC group (Fig. 7, *C* and *D*).

To confirm the regulatory role of PPIL2 in HCC cell senescence *in vivo*, frozen sections of the collected tumor tissues were subjected to SA-β-gal staining. The results showed that the PPIL2-knockdown group had a significantly higher proportion of SA-β-gal-positive areas compared to the NC group (Fig. 7*E*). Since Ki67 expression is closely associated with tumor cell proliferation and is a widely used proliferation marker, we further assessed tumor proliferation through Ki67 staining. Therefore, tumor tissues were stained with H&E to observe tumor cell morphology. Then Ki67 staining results demonstrated that Ki67 percentage were significantly reduced compared with NC mice (Fig. 7*F*). Furthermore, the knockdown group exhibited markedly reduced c-Myc expression and significantly increased p21 expression relative to the NC group (Fig. 7*F*). These results demonstrate that targeting PPIL2 effectively inhibits HCC progression and induces senescence *in vivo*.

### PPIL2 increases c-myc protein abundance independent of detectable changes in c-myc ubiquitination or protein stability

**Figure 8:**
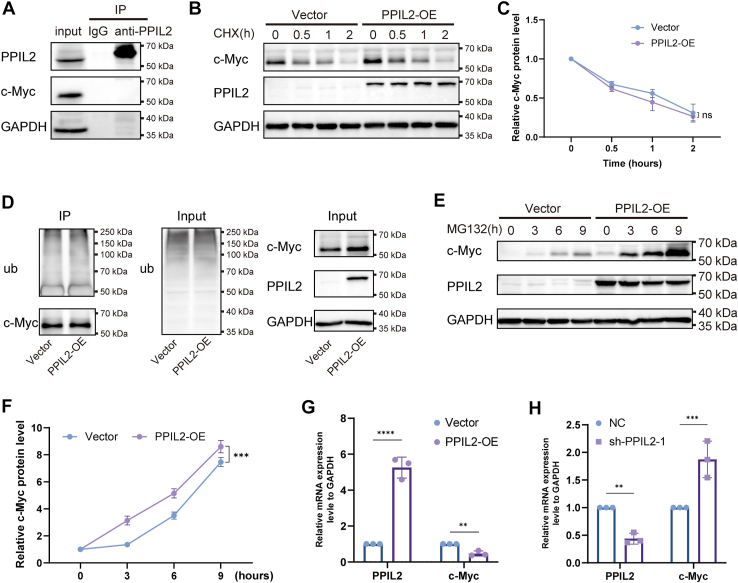
**PPIL2 positively regulates c-Myc protein abundance but does not alter detectable c-Myc ubiquitination or half-life.***A*, endogenous Co-IP assay of PPIL2 and c-Myc in Huh-7 cells. Total cell lysates were immunoprecipitated (IP) with an anti-PPIL2 antibody or IgG control, followed by immunoblotting with the indicated antibodies. *B*, representative CHX chase assay evaluating c-Myc protein stability in Huh-7 cells transfected with control vector or Flag-PPIL2. Cells were treated with 100 μg/ml cycloheximide (CHX) and harvested at the indicated time points. *C*, quantification of the CHX chase assay shown in *B*, relative c-Myc protein levels were calculated by first normalizing c-Myc to GAPDH and then normalizing each value to the corresponding 0 h c-Myc/GAPDH ratio within each group. Data are presented as mean ± S.D. from three independent biological replicates. Statistical significance was determined by two-way ANOVA with Sidak’s multiple comparisons test. ns, not significant. *D*, *in vivo* ubiquitination assay of c-Myc in Huh-7 cells transfected with vector or Flag-PPIL2. c-Myc was immunoprecipitated from whole-cell lysates using an anti-c-Myc antibody. *E*, representative immunoblot analysis of c-Myc accumulation in Huh-7 cells transfected with vector or Flag-PPIL2 and treated with MG132 for the indicated times. *F*, quantification of the MG132 accumulation assay shown in *E*, relative c-Myc protein levels were calculated by first normalizing c-Myc to GAPDH and then normalizing each value to the corresponding 0 h c-Myc/GAPDH ratio within each group. Data are presented as mean ± S.D. from three independent biological replicates. Statistical significance was determined by two-way ANOVA with Sidak’s multiple comparisons test. ∗∗∗*p* < 0.001. *G*. RT-qPCR analysis of PPIL2 and MYC mRNA levels in Huh-7 cells overexpressing PPIL2. mRNA levels were normalized to GAPDH. Data are presented as mean ± S.D. from three independent biological replicates. Statistical significance was analyzed by one-way ANOVA with Tukey’s multiple comparisons test. ∗∗*p* < 0.01; ∗∗∗∗*p* < 0.0001. *H*. RT-qPCR analysis of PPIL2 and MYC mRNA levels in Huh-7 cells following PPIL2 knockdown. mRNA levels were normalized to GAPDH. Data are presented as mean ± S.D. from three independent biological replicates. Statistical significance was analyzed by one-way ANOVA with Tukey’s multiple comparisons test. ∗∗*p* < 0.01; ∗∗∗*p* < 0.001.

Because PPIL2 is a U-box-type E3 ubiquitin ligase, we asked whether PPIL2 directly regulates c-Myc through a ubiquitin-dependent mechanism. Endogenous Co-IP failed to detect a stable PPIL2-c-Myc interaction under the conditions tested (Fig. 8*A*). Moreover, PPIL2 overexpression did not significantly alter c-Myc protein half-life in CHX chase assays (Fig. 8, *B* and *C*) or the overall polyubiquitination level of c-Myc (Fig. 8*D*). By contrast, MG132 treatment led to greater c-Myc accumulation in PPIL2-overexpressing cells than in control cells (Fig. 8, *E* and *F*), indicating that PPIL2 promotes c-Myc protein accumulation under proteasome-inhibited conditions. Notably, PPIL2 overexpression decreased MYC mRNA levels, whereas PPIL2 knockdown increased MYC mRNA expression (Fig. 8, *G* and *H*). These results indicate that PPIL2 positively regulates c-Myc protein abundance, but not through detectable direct binding, altered c-Myc ubiquitination, prolonged protein half-life, or increased MYC mRNA expression, suggesting a non-canonical and likely indirect regulatory mechanism.

## Discussion

In the present study, we systematically investigated the oncogenic role and clinical significance of PPIL2 in HCC. Bioinformatic and experimental analyses revealed that PPIL2 was markedly overexpressed in HCC and closely correlated with poor prognosis. Functionally, PPIL2 promoted HCC progression by facilitating cell cycle progression, enhancing proliferative phenotypes, and suppressing cellular senescence. We further validated that PPIL2 depletion reduced c-Myc levels and induced p21 expression, leading to cell cycle arrest and senescence—an effect partially reversed by exogenous c-Myc. Thus, we concluded that PPIL2 drives hepatocarcinogenesis by sustaining c-Myc-mediated repression of the senescence machinery, highlighting its potential as a novel prognostic biomarker and therapeutic target in HCC.

Accumulating evidence has implicated PPIL2 in cancer progression. In breast cancer, PPIL2 acts as a tumor suppressor by regulating cytoskeletal remodeling and inhibiting epithelial-mesenchymal transition (7). Conversely, in lung adenocarcinoma, PPIL2 variants contribute to early carcinogenesis by interfering with non-homologous end joining (NHEJ) DNA repair mechanisms (9). In the present study, we demonstrated that PPIL2 is specifically overexpressed in HCC tissues and correlates with dismal patient outcomes, including significantly shorter OS, DSS, and PFI. Notably, PPIL2 displayed robust diagnostic efficacy in discriminating HCC from normal liver tissues (ROC AUC = 0.873). These findings support the hypothesis that PPIL2 may serve as a robust prognostic biomarker in HCC.

Given PPIL2’s association with poor prognosis, we next explored the biological functions of PPIL2 in HCC. GSEA revealed a strong correlation between PPIL2 expression and cell cycle-related pathways. Aberrant cellular proliferation constitutes a hallmark of cancer (14). Cell cycle dysregulation drives tumor proliferation through checkpoint abnormalities that permit uncontrolled growth (15). Proper cell cycle regulation is crucial for cellular survival and proliferation, with cyclins representing key anticancer therapeutic targets (16). Flow cytometry analysis showed that PPIL2 knockdown led to a marked G0/G1 phase arrest, accompanied by a significant reduction in the S and G2/M phase populations. Consistently, functional assays demonstrated that PPIL2 silencing suppressed colony formation, metabolic activity, and DNA synthesis. Collectively, these findings establish a critical role for PPIL2 in promoting HCC proliferation and cell cycle regulation.

Currently, leveraging senescence-induced cell cycle arrest has emerged as a promising therapeutic strategy against cancer (17). Cellular senescence, characterized by permanent cell cycle exit and structural degeneration, plays a pivotal role in limiting malignant progression (18). Our data demonstrate that suppression of PPIL2 significantly induced cellular senescence in HCC, as evidenced by increased SA-β-gal activity, elevated p21 expression, and characteristic senescence-associated morphological changes. In aggressive cancers such as HCC, inducing senescence may create a critical therapeutic window by limiting rapid tumor growth. Given PPIL2 maintains HCC cell proliferation, targeting this cyclophilin-like protein to overcome senescence evasion merits further investigation as a promising therapeutic strategy.

At the molecular level, p21 inhibits cyclin-CDK complexes to induce cell cycle arrest and acts as a key effector of the senescence program (19, 20). Mechanistically, c-Myc represses p21 transcription by forming an inhibitory complex with Miz-1 at the gene promoter (11). The onset of cellular senescence is therefore tightly linked to c-Myc downregulation, which relieves this transcriptional block, leading to p21 accumulation and ultimately driving the senescent phenotype (21, 22, 23). In line with this model, our results demonstrate that PPIL2 depletion reduces c-Myc levels while concurrently upregulating p21, along with decreased expression of c-Myc target genes such as CDK4, Cyclin D1, and CDK2. Importantly, exogenous c-Myc overexpression in PPIL2-knockdown cells partially attenuated p21 induction and reversed the senescent phenotype. These findings suggest that PPIL2 sustains HCC cell survival by maintaining c-Myc-mediated suppression of the senescence machinery.

Mechanistically, our data do not support a model in which PPIL2 directly stabilizes c-Myc through ubiquitin-dependent regulation. Despite being a U-box-type E3 ubiquitin ligase, PPIL2 did not show a detectable stable interaction with c-Myc, nor did it measurably alter c-Myc ubiquitination or protein half-life. Although c-Myc accumulated to a greater extent in PPIL2-overexpressing cells during MG132 treatment, this effect is better interpreted as enhanced protein accumulation rather than direct evidence of increased c-Myc stability. Notably, MYC mRNA changed in the opposite direction, decreasing upon PPIL2 overexpression and increasing after PPIL2 knockdown. Thus, the effect of PPIL2 on c-Myc is unlikely to occur at the level of direct ubiquitin-mediated proteostasis or transcriptional upregulation of MYC. Instead, PPIL2 appears to regulate c-Myc through an indirect, non-canonical mechanism, potentially involving translational or upstream signaling control, with the reciprocal change in MYC mRNA consistent with compensatory feedback.

In summary, our study identifies PPIL2 as a pro-tumorigenic factor in HCC and indicates that PPIL2 promotes malignant progression, at least in part, through the c-Myc/p21 axis. However, the present data do not support direct ubiquitination-dependent stabilization of c-Myc by PPIL2. Instead, PPIL2 increases c-Myc protein abundance through a mechanism that is independent of detectable stable interaction, altered overall ubiquitination, prolonged protein half-life, or increased MYC mRNA expression. Future studies should focus on identifying the intermediate factor or signaling pathway through which PPIL2 enhances c-Myc protein accumulation and determining whether this effect is mediated through translational regulation, altered protein synthesis, or other indirect mechanisms.

## Experimental procedures

### Bioinformatics data and resources

The transcriptomic analysis utilized Level 3 RNA-seq data from The Cancer Genome Atlas (TCGA) database, encompassing 33 distinct human malignancies. For HCC investigations, paired RNA-sequencing datasets and clinical annotations were specifically curated from the TCGA-Liver Hepatocellular Carcinoma (TCGA-LIHC) cohort and processed according to the methods established in our prior work (24).

### Survival analysis

Kaplan-Meier estimators with log-rank test statistics were implemented to delineate survival disparities, employing the optimal expression-based stratification for PPIL2 derived *via* the surv_cutpoint algorithm (survminer v0.4.9). This non-parametric approach utilized maximally selected rank statistics to determine the biologically relevant threshold for dichotomizing cohorts into high-*versus* low-expression subgroups. Multidimensional survival assessment was performed using the survival package (v3.5–7), integrating three oncological endpoints: overall survival (OS), progression-free interval (PFI), and disease-specific survival (DSS). The optimal expression cut-off derived from maximally selected rank statistics was applied to dichotomize patients into high- and low-expression cohorts for comparative survival modeling.

### Receiver–operating characteristic curve

Receiver–operating characteristic (ROC) curves were plotted to assess the diagnostic value of PPIL2, and the areas under the ROC curve (AUC) were calculated using the pROC package. According to the classification criteria proposed by Hosmer and Lemeshow, the discriminatory performance was interpreted as follows: an AUC of 0.5 indicates no discrimination, 0.7 ≤ AUC < 0.8 suggests acceptable discrimination, 0.8 ≤ AUC < 0.9 indicates excellent discrimination, and an AUC ≥ 0.9 represents outstanding discrimination (25).

### Logistic regression analysis

The single-gene logistic regression analysis was performed to evaluate the association between PPIL2 expression and the outcomes of interest. Statistical analyses were conducted using R (version 3.6.3) with basic R packages for data processing, statistical modeling, and visualization. PPIL2 expression levels were treated as the dependent variable, while independent variables were dichotomized into "Low" and "High" categories based on median expression level of PPIL2. Subgroup analysis was performed by stratifying the population according to the median PPIL2 expression level.

### Gene set enrichment analysis

Based on the TCGA-LIHC database, samples were stratified into two groups according to the expression level of PPIL2: a high-expression group and a low-expression group. Differential gene expression analysis was performed between the two groups to calculate the fold change (FC) of each gene. Subsequently, Gene set enrichment analysis (GSEA)was conducted to identify enriched biological pathways.

GSEA was performed to identify enriched biological pathways in the dataset using the R package clusterProfiler (version 4.4.4) (26). Gene symbols in the input dataset were converted to Entrez IDs using the org.Hs.*e.g.,*.db R package, ensuring compatibility with downstream analysis tools. The reference gene set "c2.cp.all.v2022.1.Hs.symbols.gmt" was obtained from the Molecular Signatures Database (MSigDB Collections) and imported *via* the msigdbr R package (27). This curated collection includes canonical pathways from diverse sources such as KEGG, Reactome, and BioCarta. The GSEA was conducted in clusterProfiler with default parameters. Ranked gene lists were generated based on pre-defined metrics. Enrichment scores were calculated through 10,000 permutations to assess statistical significance, with pathways meeting p.adjust <0.05 considered statistically enriched.

### Cell culture

HEK 293T cell line and hepatocellular carcinoma-derived Huh-7 and Hep3B cells were purchased from Shanghai Cell Bank, Chinese Academy of Science. Cells were authenticated by short tandem repeat (STR) profiling and were confirmed to be mycoplasma-free. These cells were cultured in Dulbecco's Modified Eagle Medium (DMEM) containing 10% (v/v) heat-inactivated fetal bovine serum (FBS) and 1% penicillin-streptomycin (10,000 U/ml penicillin, 10 mg/ml streptomycin). All cultures were maintained in a humidified incubator with precisely controlled atmospheric conditions (37 °C, 5% CO_2_) with daily monitoring of confluence and medium replenishment. For experimental standardization, cells in logarithmic-phase growth (80–90% confluence) were dissociated, demonstrating using 0.25% trypsin-EDTA solution and subcultured at predetermined densities to maintain phenotypic stability across replicates.

### Lentivirus infection

For targeted PPIL2 knockdown, two shRNA sequences (sh-PPIL2-1: TCTCAATTCTTCATCACGTTT; sh-PPIL2-2: GCTAAATATCAAGGCCAAGAA) were inserted into the linearized pLV3-U6-MCS-shRNA vector using restriction enzyme-based subcloning. For PPIL2 overexpression, the full-length coding sequence was PCR-amplified using NCBI GenBank reference sequences as templates and cloned into the pLV3-CMV-MCS expression vector with a C-terminal FLAG tag. Lentiviral particle preparation and the screening of stable cell lines were carried out according to the method reported in our prior study (28).

### Western blotting analysis

Protein lysates were extracted from Huh-7 cells using RIPA buffer (Beyotime) supplemented with protease and phosphatase inhibitor cocktails. Protein concentrations were determined using the Bradford protein assay kit (Beyotime) followed by denaturation at 98 °C for 10 min. Samples were resolved by 10% SDS-PAGE and subsequently transferred to polyvinylidene difluoride (PVDF) membranes. Membranes were blocked with protein-free rapid blocking buffer (Epizyme Biotech) for 30 min at room temperature, then incubated overnight at 4°C with the following primary antibodies: PPIL2 (Proteintech, 12590-2-AP; 1:1000), CDK2 (Upingbio, YP-Ab-16699; 1:2000), CDK4 (Upingbio, YP-Ab-16791; 1:2000), Cyclin D1 (Upingbio, YP-Ab-16725; 1:2000), Cyclin E1 (Upingbio, YP-Ab-16728; 1:2000), PCNA (Proteintech, 10205-2-AP; 1:1000), p-STAT3 (Abmart, T56566; 1:1000), STAT3 (Abmart, T55292; 1:1000), c-Myc (Huabio, 0912–2; 1:1000), and GAPDH (Proteintech, 10494-1-AP; 1:5000). Following three washes with TBST, membranes were incubated with horseradish peroxidase (HRP)-conjugated secondary antibodies (Proteintech, SA00001–2; 1:6000) for 1 h at room temperature. Protein signals were detected using enhanced chemiluminescence (ECL) substrate (Proteintech) and quantified by densitometric analysis with ImageJ software (National Institutes of Health).

### Colony formation assay

Huh-7 cells with stable PPIL2 knockdown or overexpression were resuspended and plated at clonal densities (300 cells/well in 12-well plates or 700 cells/well in 6-well plates). Cells were maintained in complete medium for 14 days under standard culture conditions to allow colony formation. Following methanol fixation with 4% paraformaldehyde (PFA, 15 min at room temperature), colonies were stained with 1% (w/v) Crystal violet solution (10 min) and extensively washed with phosphate-buffered saline (PBS) to remove background staining. Quantification was performed by counting cell clusters containing ≥50 cells as statistically valid colonies.

### CCK8 assay

Huh-7 cells (3000 cells/well) were plated in 96-well plates post-transfection and cultured under standard conditions. Cell viability was assessed at 24-, 48-, 72-, and 96-h intervals using the CCK-8 assay (APE x BIO Technology). Specifically, 10 μl of CCK-8 reagent was added to each well, followed by 2 h incubation. Absorbance at 450 nm was quantified using a BioTek Synergy H1 microplate reader (Agilent Technologies).

### EdU staining

Cell proliferation was assessed using the BeyoClick EdU Cell Proliferation Kit with Alexa Fluor 594 (Beyotime Biotechnology, C0078S) according to the manufacturer's protocol. Briefly, transfected cells were fixed with 4% paraformaldehyde and then washed with PBS. Next, cells were incubated with EdU working solution for 2 h and stained with DAPI at room temperature for 15 min. Cells were observed under a fluorescent microscopy (Olympus, Tokyo, Japan).

### Cell cycle

Cell cycle distribution was analyzed using the Cell Cycle and Apoptosis Analysis Kit (Beyotime Biotechnology, C1052). Huh-7 cells were seeded in 6-well plates at a density of two × 10^5^ cells/well and synchronized by serum starvation for 24 h. At 60% confluence, cells were harvested by trypsinization, washed twice with ice-cold PBS, and fixed in 70% ethanol (pre-chilled to −20 °C) overnight at 4 °C. Fixed cells were pelleted by centrifugation (300*g*, 5 min), rehydrated in PBS, and treated with RNase A at 37 °C for 30 min to eliminate RNA interference. DNA was stained with propidium iodide (PI) for 15 min in the dark. The cell cycle was detected using flow cytometry. Data analysis was performed using FlowJo software (BD Biosciences) to quantify G0/G1, S, and G2/M phase distributions.

### Immunofluorescence staining

A cover slide was placed into each well of a 24-well plate, and 1 × 10^4^ cells were seeded per well for a 48h culture. The cells were then fixed with 4% paraformaldehyde (Biosharp, BL539A) for 30 min, followed by permeabilization and blocking with 3% bovine serum albumin (BSA) for another 30 min. After blocking, the cells were incubated overnight at 4 °C with primary antibodies diluted at 1:200. Subsequently, the cells were incubated for 1 h at room temperature with a mixture of fluorescently-labeled secondary antibodies (1:500) and phalloidin (Beyotime Biotechnology, C2209S; 1:200) to visualize F-actin. Nuclei were counterstained with DAPI (Beyotime Biotechnology, P0131). Finally, the coverslips were mounted with an anti-fade mounting medium to minimize fluorescence quenching.

### SA-β-gal staining

SA-β-gal (Senescence-associated β-galactosidase) staining was performed using the Senescence β-Galactosidase Staining Kit according to the manufacturer's instructions. Cells exhibiting perinuclear blue precipitates were identified as SA-β-gal-positive cells. The senescence index was calculated as follows: (number of SA-β-gal^+^ cells/Total cells) × 100%. In tumor tissues, SA-β-gal-positive areas were quantified by threshold-based pixel density analysis using ImageJ software. Three independent biological replicates were established per experimental group. From each biological replicate, five non-overlapping fields of view were randomly captured using systematic random sampling *via* inverted microscope (Olympus Corporation, CKX53).

### Human tumor specimens

Human HCC tumor specimens and corresponding nontumor specimens utilized in this study, comprising both fresh tissue samples and paraffin-embedded sections, were obtained from the Pathology Department of the Affiliated Hospital of Southwest Medical University. All specimens were collected following written informed consent for diagnostic confirmation of HCC in accordance with the World Health Organization diagnostic criteria. The experimental protocol was reviewed and approved by the Institutional Ethics Committee of Southwest Medical University (Approval No. KY2025340).

### Tumor xenograft experiments

All experimental procedures were conducted in strict accordance with the National Institutes of Health Guide for the Care and Use of Laboratory Animals following approval by the Southwest Medical University Institutional Animal Care and Use Committee (Approval No. swmu20250610). Male BALB/c nude mice (4-week-old, 16–20*g* body weight) were procured from Chongqing Tenxin Biotechnology and acclimatized for 7 days in specific pathogen-free (SPF) conditions with controlled environmental parameters: 12/12 h light/dark cycle, 20 to 22 °C temperature, 50 to 60% humidity, and ad libitum access to autoclaved rodent chow and UV-sterilized water.

Following computer-generated randomization, 18 weight-matched mice were stratified into three experimental cohorts (n = 6/group). For tumor xenograft establishment, single-cell suspensions (1 × 10^6^ cells/ml in PBS) with >95% viability (trypan blue exclusion) were prepared through enzymatic dissociation and implanted subcutaneous tissue of the right upper limb axilla using insulin syringe. Tumor progression was monitored every 3 days using digital vernier calipers, with volumetric calculations employing the modified ellipsoid formula: TV (mm^3^) = 0.5 × d^2^ × D (d: minor axis; D: major axis). Tumors were harvested 28 days after implantation. The tumors were weighed immediately; samples were fixed in 4% paraformaldehyde for 24 h for histopathology or snap-frozen in liquid nitrogen for frozen section and molecular analyses.

### H&E and immunohistochemical staining

Prior to immunohistochemical investigation, tumor specimens were processed through standardized histopathological protocols. For hematoxylin and eosin (H&E) staining, 4 μm-thick tissue sections were processed through the following steps: deparaffinization, rehydration, nuclear staining with hematoxylin, cytoplasmic counterstaining with eosin, dehydration through an ethanol series, and final mounting with a coverslip. Histomorphological evaluation was conducted by two board-certified pathologists. Hematoxylin and eosin staining were used to label the nucleus and cytoplasm, respectively, enabling the observation of pathological features of tumor tissues.

The paraffin-embedded tissue sections were processed following the protocol of the immunohistochemical detection kit (Proteintech, PK10006). Initially, the sections were deparaffinized to water. Antigen retrieval was then performed using citric acid-based retrieval solution under heated conditions. Endogenous peroxidase activity was quenched to prevent nonspecific staining. Sections were subsequently blocked with 5% BSA to reduce background staining. Primary antibody incubation was carried out overnight at optimal conditions, followed by a 1-h incubation with the appropriate secondary antibody. Chromogenic detection was achieved using DAB substrate, and nuclei were counterstained with hematoxylin. Finally, the sections were dehydrated, mounted, and examined under a light microscope for analysis.

### Quantitative RT-PCR

Total RNA was extracted using AG RNAex Pro Reagent (Accurate Biotechnology, AG21101, China) according to the manufacturer’s instructions. Subsequently, cDNA synthesis was performed using HisyGo RT Red SuperMix (Vazyme, RT101–01). Quantitative real-time PCR (RT-qPCR) was conducted on the Applied Biosystems QuantStudio 5 Real-Time PCR System with SYBR qPCR Master Mix (Vazyme, Q312–02, China). GAPDH served as the endogenous control for normalization. All reactions were performed in technical triplicates. Relative gene expression levels were determined using the comparative ΔΔCt method. The specific primer sequences used for qPCR analysis were as follows: PPIL2 (forward: 5′-TTTGTCTACCCAGTCTGCA

CTC-3′, reverse: 5′-TCCCGTACTTCTTAAGCCATGG-3′), c-Myc (forward: 5′-GGCTCCTGGCAAAAGGTCA-3′, reverse: 5′-CTGCGTAGTTGTGCTGATGT-3′) and GAPDH (forward: 5′-GGAGCGAGATCCCTCCAAAAT-3′, reverse: 5′-GGCTGTTGTCATACTTCTCATGG-3′).

### Co-immunoprecipitation (Co-IP) and Western blotting

Cells were lysed in ice-cold NP-40 buffer supplemented with protease inhibitors. After 30 min of incubation on ice, lysates were cleared by centrifugation (12,000*g*, 15 min, 4 °C). For each IP, total protein was incubated with specific antibodies or control IgG overnight at 4 °C, followed by 2h of incubation with Protein A/G magnetic beads. The beads were washed five times with lysis buffer, and bound proteins were eluted by boiling in 1× SDS-PAGE loading buffer at 95 °C for 10 min. Immunoprecipitates and whole-cell lysates (5% input) were resolved by SDS-PAGE, transferred to PVDF membranes, and analyzed by immunoblotting.

### *In vivo* ubiquitination assay

Cells transfected with Flag-PPIL2 or empty vectors were treated with 10μM MG132 for 6h before harvesting. To ensure the detection of direct c-Myc ubiquitination, cells were lysed in ice-cold NP-40 buffer supplemented with protease inhibitors. Lysates were boiled at 95°C for 10 min and subsequently diluted 10-fold with SDS-free lysis buffer. The diluted lysates were subjected to immunoprecipitation using an anti-c-Myc antibody and Protein A/G magnetic beads at 4 °C. After four washes, the immunoprecipitates were eluted by boiling in 1 × SDS-PAGE loading buffer and analyzed by immunoblotting. Polyubiquitinated c-Myc was detected with anti-Ub antibody.

### Statistics and reproducibility

Statistical analyses were conducted utilizing R (version 4.1.1) and GraphPad Prism 9.0.0 software. Continuous variables were expressed as mean ± SD, with between-group comparisons performed using Student's *t* test (two groups) or one-way ANOVA with Tukey's multiple comparisons test or Two-way ANOVA with Sidak's multiple comparisons test (multiple groups). Bivariate correlations were examined through Spearman's rank correlation coefficient. Survival outcomes were compared between groups using Kaplan-Meier curves with log-rank testing for statistical significance. All statistical tests employed a significance threshold of *p* < 0.05.

### Data availability

Transcriptomic data for this study were obtained from The Cancer Genome Atlas (TCGA) through the National Cancer Institute's Genomic Data Commons (GDC) portal (https://portal.gdc.cancer.gov/). The data that support the findings of this study are available on request from the corresponding author.

## Supporting information

This article contains supporting information.

## Conflict of interest

The authors declare that they have no conflicts of interest with the contents of this article.
