# Supplementary material for: PPIL2 suppression induces cellular senescence and inhibits proliferation in hepatocellular carcinoma via c-myc/p21 axis
Source: J Biol Chem. 2026 May 6;302(6):113109. doi: 10.1016/j.jbc.2026.113109 (PMC13254592; doi:10.1016/j.jbc.2026.113109)
Supplement: Figure S1 [file mmc1.pdf]

Supplementary Figure 1

Title: PPIL2 suppression induces cellular senescence and inhibits proliferation in hepatocellular carcinoma via c-Myc/p21 axis

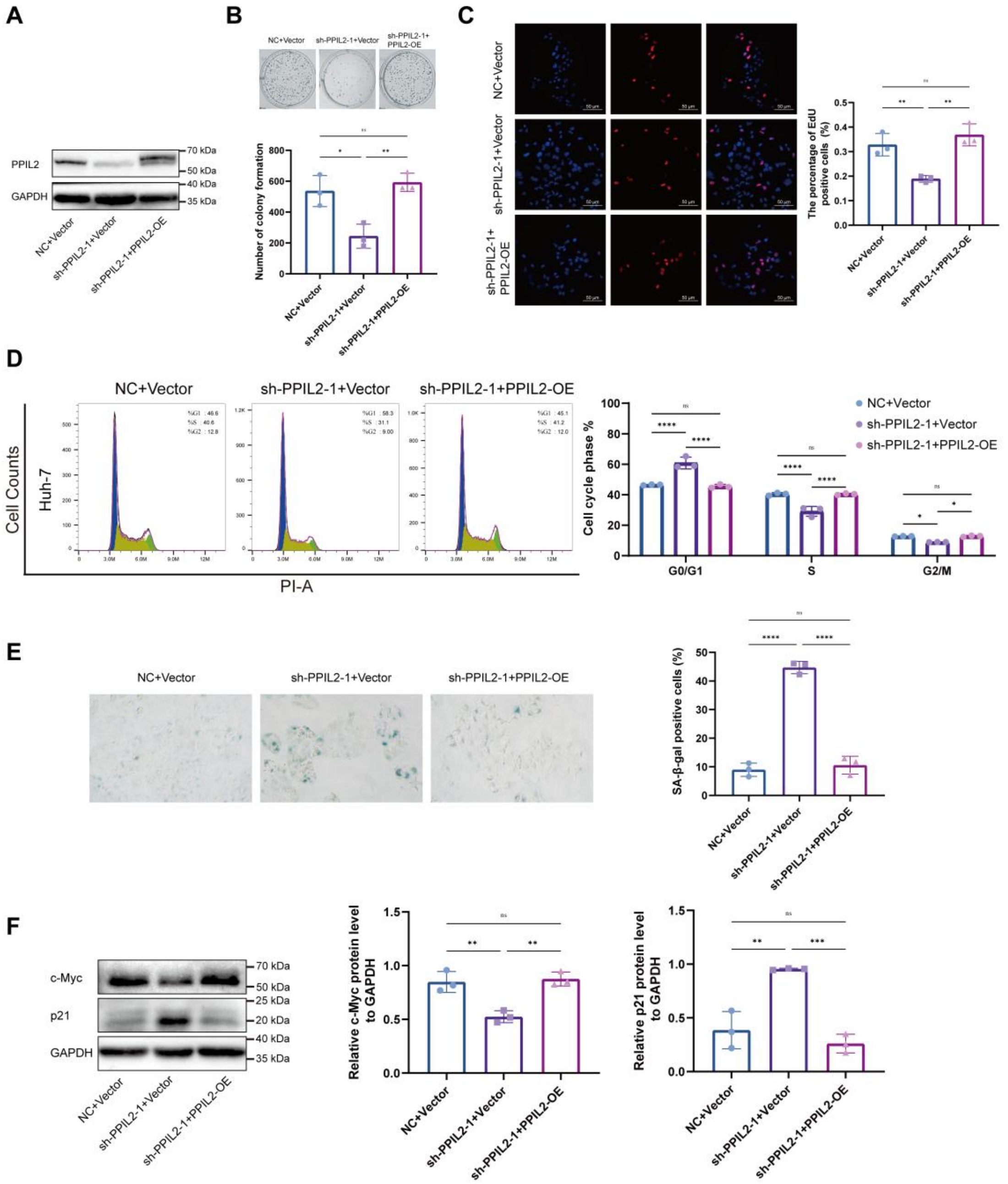

Supplementary Figure 1: Rescue experiments confirming the on-target effects of PPIL2 knockdown. *A*. Western blot validation of PPIL2 knockdown or overexpression efficiency in Huh-7 cells transfected with PPIL2 shRNA, PPIL2 shRNA plus Flag-tagged PPIL2 cDNA (rescue), or empty vector. Protein expression was normalized to the housekeeping gene GAPDH. *B*. Colony-formation assay to

evaluate the proliferation capacity of Huh-7 cells in the aforementioned groups. The number of colonies is expressed as the mean ± S.D. from 3 independent biological replicates. Statistical significance was analyzed by one-way ANOVA with Tukey's multiple comparisons test. *p<0.05; **p<0.01; ns, not significant. *C*. EdU assay to assess the proliferation capacity of Huh-7 cells in the aforementioned groups. Scale bar: 50 μm. The percentage of EdU positive cells is expressed as the mean ± S.D. from 3 independent biological replicates. Statistical significance was analyzed by one-way ANOVA with Tukey's multiple comparisons test. **p<0.01; ns, not significant. *D*. Flow cytometry analysis to determine the cell cycle distribution of Huh-7 cells in the aforementioned groups. Data are mean ± S.D. (n=3). Two-way ANOVA with Sidak's multiple comparisons test. *p<0.05; ns, not significant. *E*. SA-β-Gal staining to detect cellular senescence in Huh-7 cells in the aforementioned groups, and the percentage of SA-β-gal-positive cells was quantified. Scale bar: 50 μm. Data are mean ± S.D. (n=3). Statistical significance was determined by one-way ANOVA with Tukey's multiple comparisons test. ****p<0.0001; ns, not significant. *F*. Western blot analysis of the expression levels of downstream effector proteins c-Myc and p21 in Huh-7 cells after PPIL2 knockdown or rescue. Protein expression was normalized to the housekeeping gene GAPDH. Data are mean ± S.D. (n=3). Statistical significance was determined by one-way ANOVA with Tukey's multiple comparisons test. **p<0.01; ***p<0.001; ns, not significant.
