# Supplementary material for: PPIL2 suppression induces cellular senescence and inhibits proliferation in hepatocellular carcinoma via c-myc/p21 axis
Source: J Biol Chem. 2026 May 6;302(6):113109. doi: 10.1016/j.jbc.2026.113109 (PMC13254592; doi:10.1016/j.jbc.2026.113109)

Original Western Blots

Title: PPIL2 Suppression Induces Cellular Senescence and Inhibits Proliferation in Hepatocellular Carcinoma via c-Myc/p21 Axis

Figure 1H

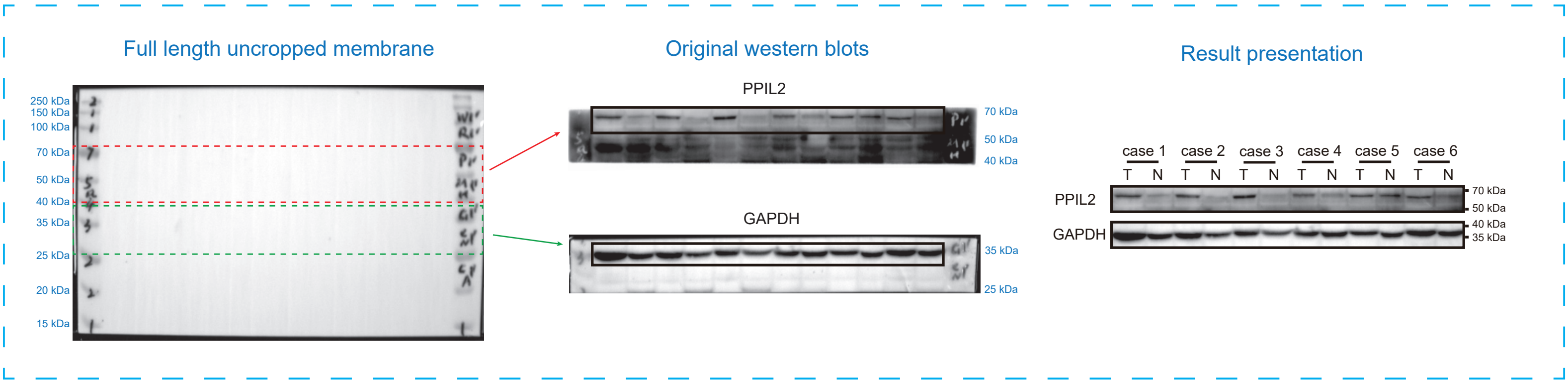

Figure 2A

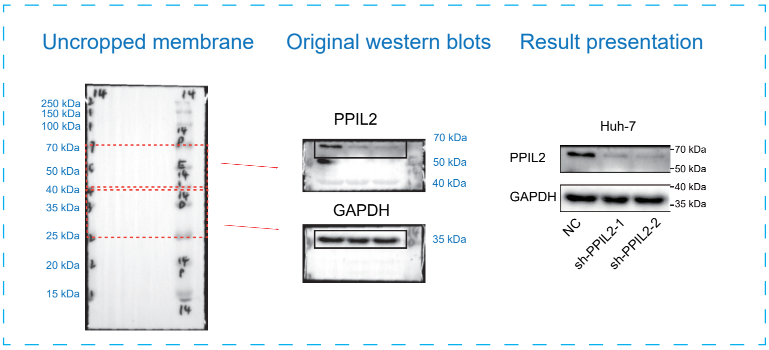

Figure 2B

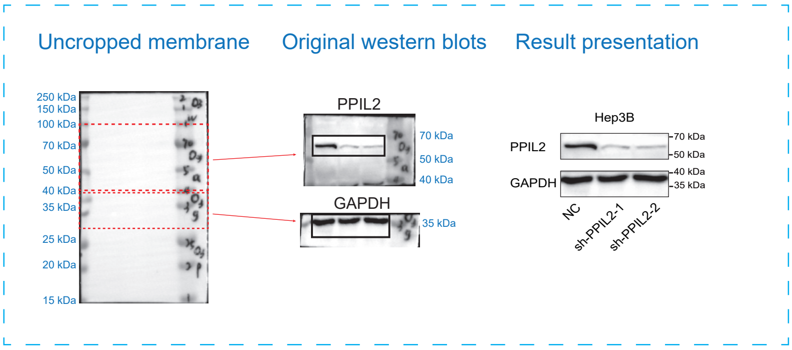

Figure 4B

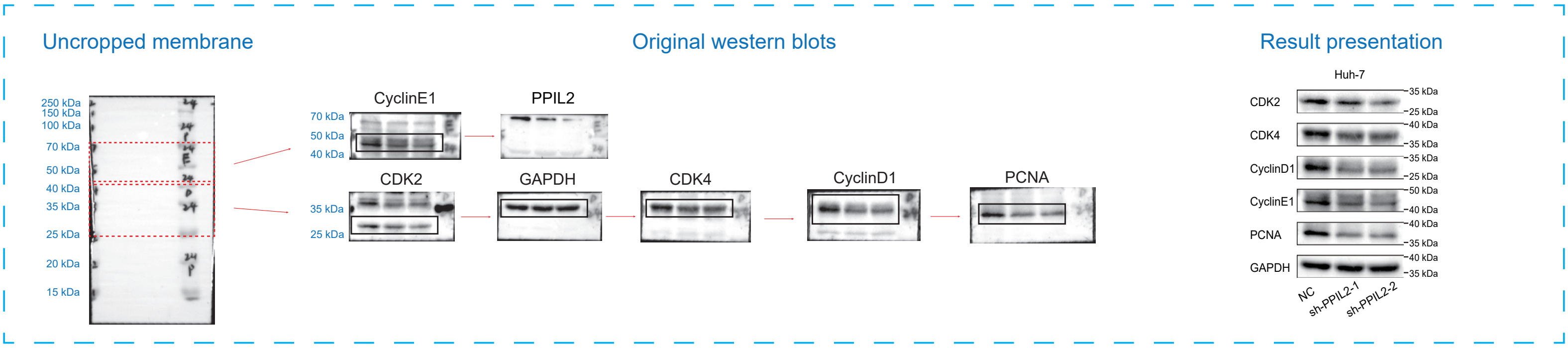

Figure 4C

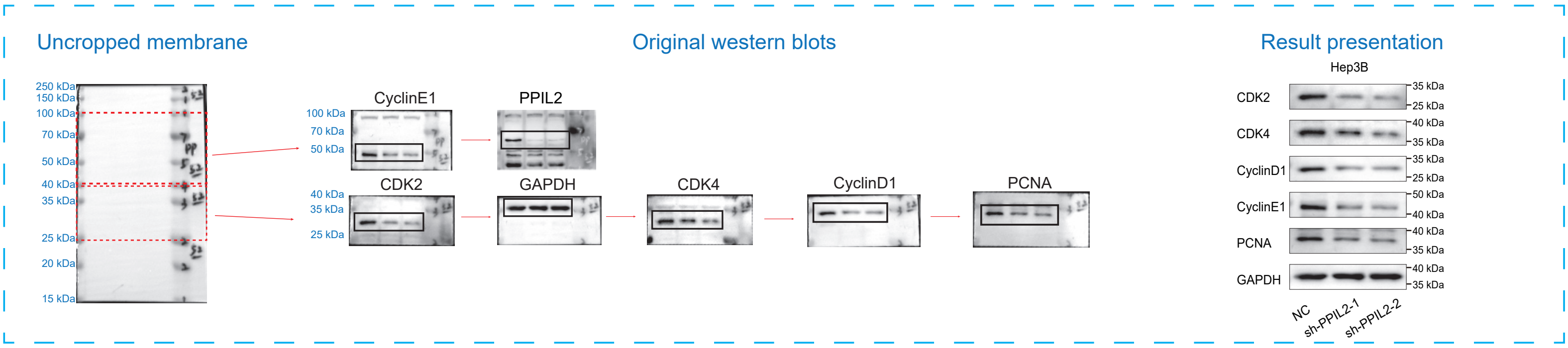

Figure 5E

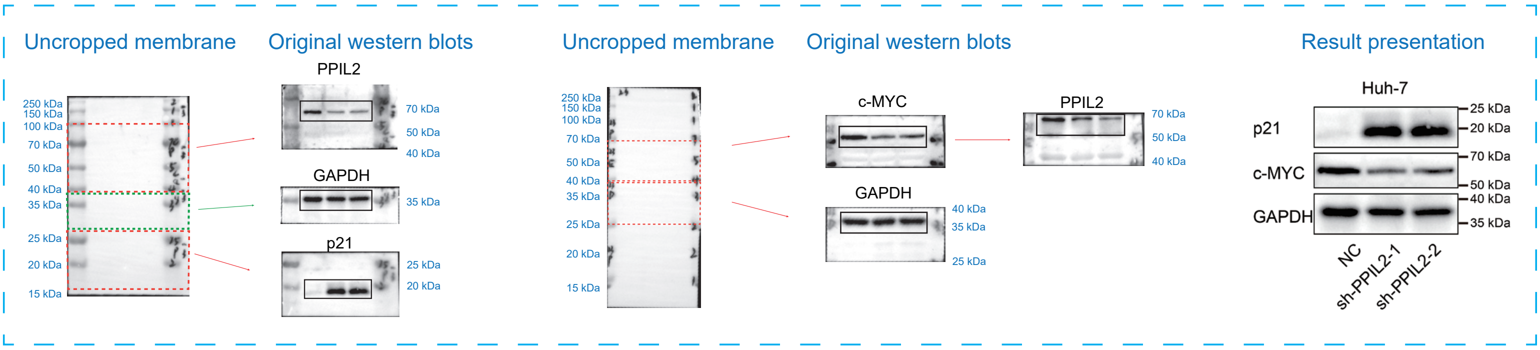

Figure 5F

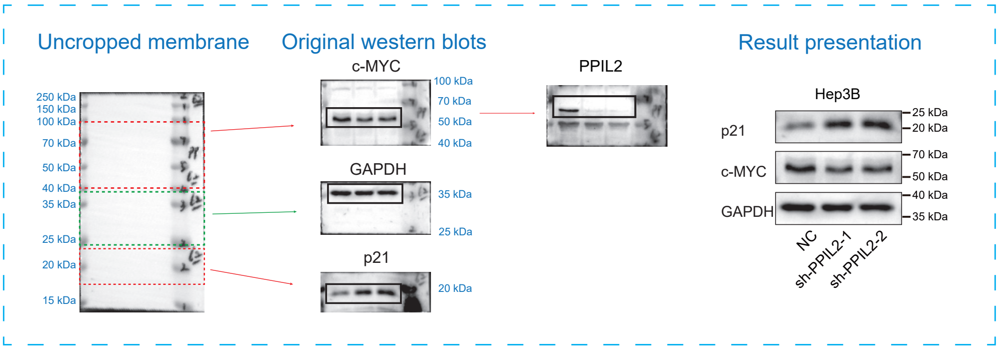

Figure 6A

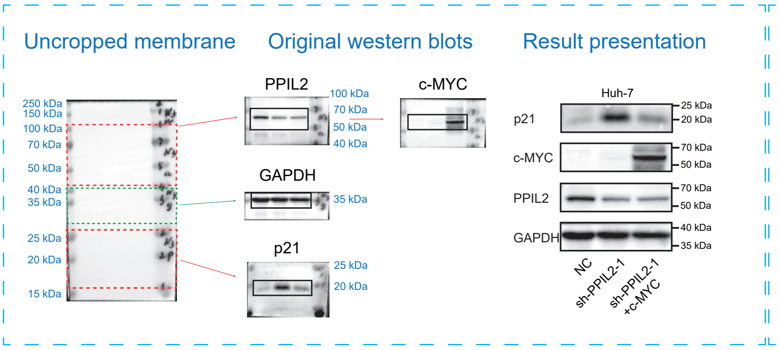

Figure 6B

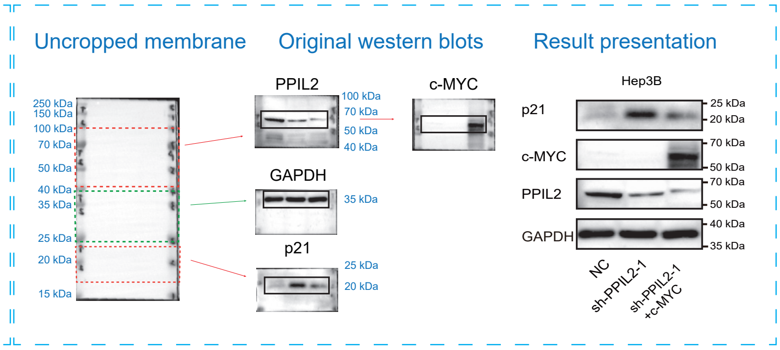

Figure 8A

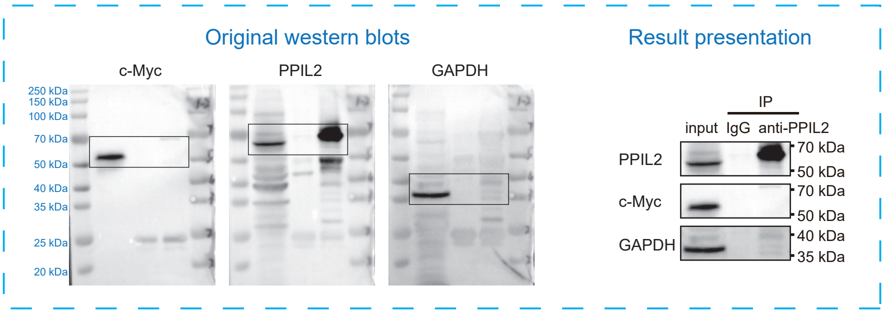

Figure 8B

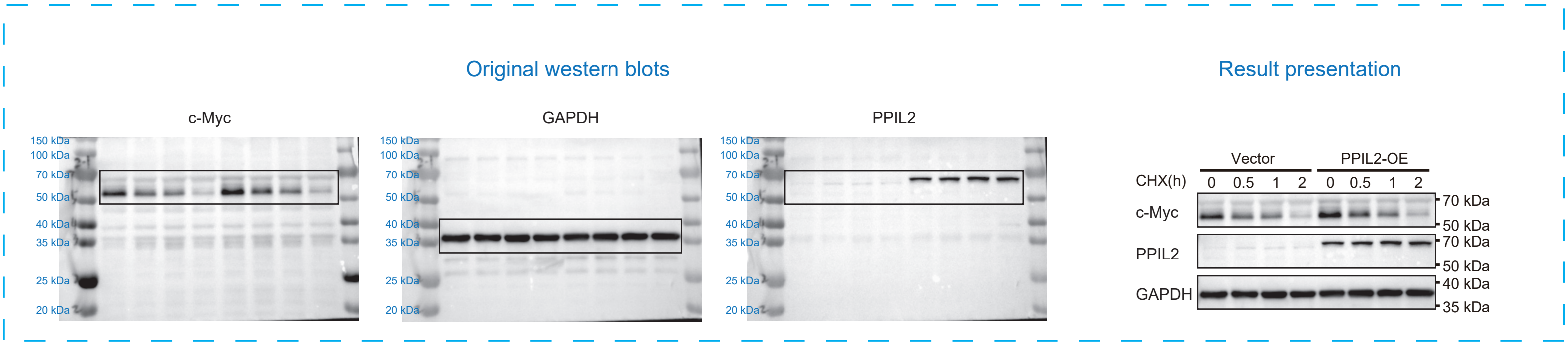

Figure 8D

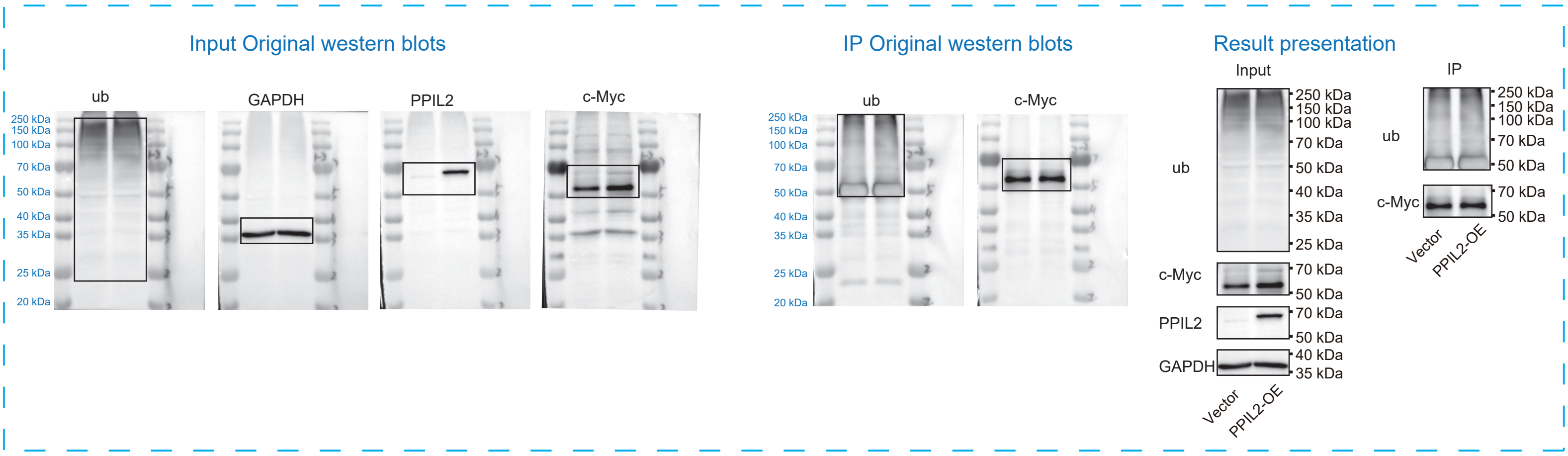

Figure 8E

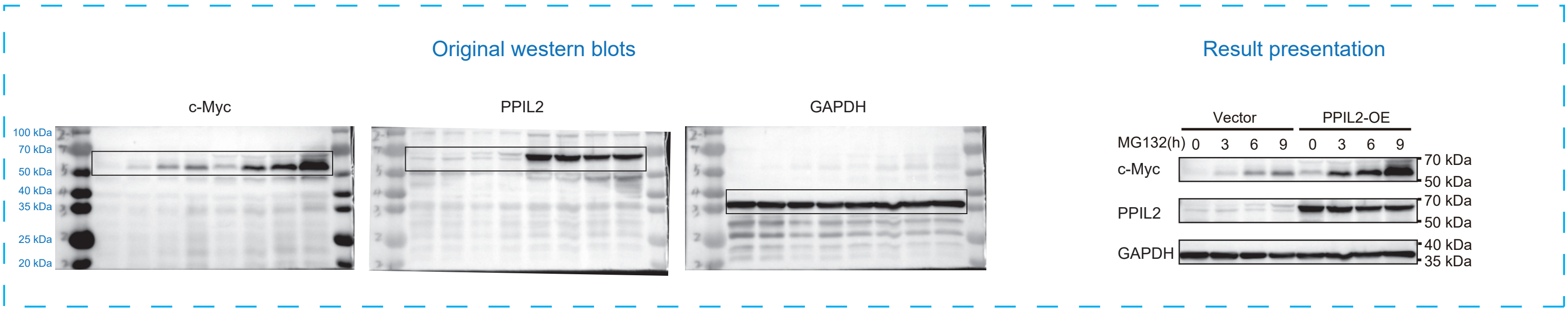

Supplementary Figure 1A

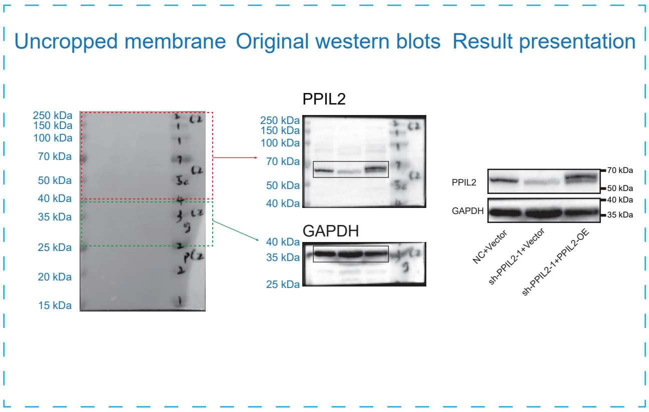

Supplementary Figure 1F

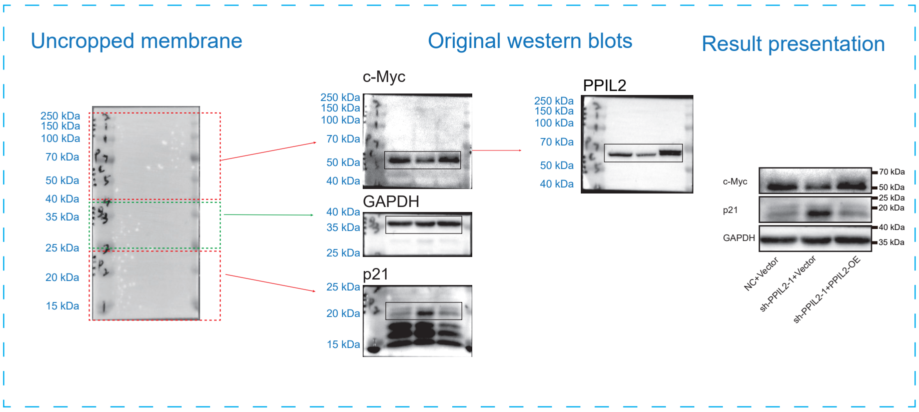

Supplement: Supplementary material [file mmc2.pdf]
